# Activation of locus coeruleus heme oxygenase-carbon monoxide pathway promoted an anxiolytic-like effect in rats

**DOI:** 10.1590/1414-431X20165135

**Published:** 2016-04-08

**Authors:** P.G. Carvalho-Costa, L.G.S. Branco, C.R.A. Leite-Panissi

**Affiliations:** 1 Programa de Pós-Graduação em Psicobiologia, Faculdade de Filosofia, Ciências e Letras de Ribeirão Preto, Universidade de São Paulo, Ribeirão Preto, SP, Brasil; 2 Departamento de Morfologia, Fisiologia e Patologia Básica, Faculdade de Odontologia de Ribeirão Preto, Universidade de São Paulo, Ribeirão Preto, SP, Brasil

**Keywords:** Heme oxygenase, Carbon monoxide, Locus coeruleus, Anxiety, Elevated plus maze, Light/dark box

## Abstract

The heme oxygenase-carbon monoxide pathway has been shown to play an important role in many physiological processes and is capable of altering nociception modulation in the nervous system by stimulating soluble guanylate cyclase (sGC). In the central nervous system, the locus coeruleus (LC) is known to be a region that expresses the heme oxygenase enzyme (HO), which catalyzes the metabolism of heme to carbon monoxide (CO). Additionally, several lines of evidence have suggested that the LC can be involved in the modulation of emotional states such as fear and anxiety. The purpose of this investigation was to evaluate the activation of the heme oxygenase-carbon monoxide pathway in the LC in the modulation of anxiety by using the elevated plus maze test (EPM) and light-dark box test (LDB) in rats. Experiments were performed on adult male Wistar rats weighing 250-300 g (n=182). The results showed that the intra-LC microinjection of heme-lysinate (600 nmol), a substrate for the enzyme HO, increased the number of entries into the open arms and the percentage of time spent in open arms in the elevated plus maze test, indicating a decrease in anxiety. Additionally, in the LDB test, intra-LC administration of heme-lysinate promoted an increase on time spent in the light compartment of the box. The intracerebroventricular microinjection of guanylate cyclase, an sGC inhibitor followed by the intra-LC microinjection of the heme-lysinate blocked the anxiolytic-like reaction on the EPM test and LDB test. It can therefore be concluded that CO in the LC produced by the HO pathway and acting via cGMP plays an anxiolytic-like role in the LC of rats.

## Introduction

The exposure of animals to dangerous situations is potentially effective in eliciting responses characteristic of fear and anxiety. Because there appears to be a correlation between human defensive behavior and fear- and anxiety-related defensive patterns in non-human mammals (1), many studies have used animal models to study the neural substrates involved in the modulation of fear and anxiety in humans. In particular, the elevated plus maze (EPM) is an animal model where the behavioral repertoire of rodents is used to detect effects on anxiety, and it has been proven to be bidirectionally sensitive to manipulations of anxiety (2).

It has been established that the neural substrates responsible for the modulation of defensive behaviors, aversive reactions, and emotional states fundamentally consist of the amygdala, hypothalamus, and periaqueductal gray matter. However, other structures are also related to the expression of the emotional responses; in particular, the noradrenergic system of the locus coeruleus (LC), which is a system closely correlated with attentional states, sleep/wakefulness cycles, learning and memory, reproduction, emotional behaviors and stressful situations, as well as modulation of fear and anxiety (3). In fact, stressful situations, including negative emotions, such as anxiety and/or fear, promote an increase in noradrenaline release in distinct brain areas, in particular in the hypothalamus, amygdala, and LC (4).

Neurochemistry research has shown that various systems of neurotransmitters are involved in fear and anxiety modulation, such as gamma-aminobutyric acid (GABA), serotonin, norepinephrine and glutamate (5,6). In addition, a growing number of studies have given support that nitric oxide (NO) and carbon monoxide (CO) can modulate emotional and autonomic responses related with stress (6,7). In this way, both CO and NO exert a stimulatory influence on the acute adrenocorticotropic hormone (ACTH) response to physical-emotional stressors. CO and NO share similar properties that can activate soluble guanylate cyclase (sGC), resulting in the complex regulation of 3′-5′-guanosine monophosphate (cGMP) (8,9). Likewise, Quock and Nguyen (10) demonstrated that systemic pretreatment with L-NOARG (an inhibitor of NO synthase) is able to block the anxiolytic effect of chlordiazepoxide in mice submitted to the EPM, indicating that NO may exert an anxiolytic effect in mice.

The activity of CO depends on the heme oxygenase (HO) enzyme that catalyzes the conversion of heme to CO, heme iron, and biliverdin (9). Two forms of HO, HO-1 and HO-2, have been identified, and a third form, HO-3, may be present in rats (11,12). In the central and peripheral nervous systems, HO-2 seems to be responsible for most of the HO activity and is expressed in both glial and neuronal cells (13), as well as cell layers in the olfactory bulb, hippocampus, cerebellum, spinal cord, and LC (9). It is important to note that LC displays elevated expression of HO-2, whereas the expression of nitric oxide synthase (responsible for the NO formation) is lower (9,14). Thus, it is suggested that CO participates in the modulation of the functions performed by this structure. Previous studies have demonstrated that the endogenous HO-CO-cGMP pathway in the LC is critical to modulation of thermal response during development of endotoxin fever, as well as during hypothermic response to restraint-stress (7,15). However, the role of the HO-CO-cGMP pathway in the other functions modulated by the LC remains to be clarified, including its role in emotional behavior. Furthermore, previous studies have demonstrated a marked increase of c-fos expression (16) and HO-2 expression (9) in LC after stressful stimuli. Therefore, the present study was designed to investigate whether the HO-CO pathway of the LC can modulate emotional behavior. To this end, we investigated whether the microinjection of zinc deuteroporphyrin 2,4-bis glycol (ZnDPBG, an HO inhibitor), heme-lysinate (substrate overload) or the selective inhibitor of soluble guanylate cyclase (1H-(1,2,4) oxadiazolo [4,3-a] quinoxaline-1-one (ODQ)) into the LC in different groups of rats produces alterations in emotional behavior as assessed by the elevated plus maze test (EPM) and light-dark box test (LDB) in rats.

## Material and Methods

### Animals

Experiments were performed on adult male Wistar rats weighing 250-300 g, (n=182) obtained from the animal facility of the Universidade de São Paulo, Ribeirão Preto, SP, Brazil. Animals were housed in a temperature-controlled room (24±1°C) and in a 12-h light/dark cycle (lights on at 6:00 am) with food and water *ad libitum*. The experiments were carried out in compliance with the recommendations of the Sociedade Brasileira de Ciência em Animais de Laboratório and with the approval of the Animal Care and Use Committee of the Universidade de São Paulo, Brazil at the Ribeirão Preto campus (Protocol #09.1.606.53.7). All efforts were made to minimize animal suffering.

### Surgical procedures

The rats were anesthetized by intramuscular injection of ketamine (100 mg/kg, União Química Farmacêutica Nacional S.A., Brazil) plus xylazine (10 mg/kg, Calier S.A., Spain) and placed in a stereotaxic apparatus (David-Kopf Instruments, USA) with the incisive bar at 0 mm and inclination of vertical stereotaxic bar at 15° (17). One stainless-steel guide cannula (0.6 mm o.d. and 15 mm in length) was inserted 1 mm above the right LC region (coordinates: anterior: 3.4 mm from lambda, lateral: 1.2 mm from midline, dorsal: 5.8 mm from the skull surface). As one of the experiments required two microinjections, animals designated to be used in this experiment were implanted with a second cannula (0.6 mm o.d. and 12 mm in length) in the right lateral cerebral ventricle (coordinates: anterior -1.0 mm, lateral -1.6 mm from midline, dorsal 3.2 to 3.7 mm from the skull surface); incisive bar at -3.3 mm. The displacement of the meniscus in a water manometer ensured correct positioning of the cannula in the lateral ventricle. The cannulas were fixed to the skull by means of self-polymerizing resin and an additional anchoring screw. A tight-fitting stylet was kept inside the guide cannula to prevent occlusion. After surgery, the rats received a subcutaneous injection of the anti-inflammatory and analgesic Banamine (10 mg/mL flunixin meglumine; Schering-Plough, USA) and an antibiotic (benzyl penicillin 160,000 U/kg, Fort Dodge, Brazil, administered intramuscularly). The experiments were initiated 1 week after surgery.

### Drugs and microinjection procedure

The non-selective HO inhibitor ZnDPBG, at the doses 5, 50 and 200 nmol/0.1 µL) and hemin were obtained from Porphyrin Products (USA). ZnDPBG was dissolved in 50 mM/L of Na_2_CO_3_ and stored in the dark. Hemin was used to prepare heme-lysinate (at the doses 150, 300 and 600 nmol/0.1 µL), a HO-CO-cGMP pathway inducer. Heme-free preparations were used as amino acid (L-lysine) vehicle control solutions. The vehicle of heme-lysinate consisted of L-lysine (14.2 µmol/mL), ethanol (5%), propylene glycol (40%) and sterile water (55%). The sGC inhibitor 1H-(1,2,4) oxadiazolo [4,3-a] quinoxaline-1-one (ODQ, 1.3 nmol/1.0 µL) was purchased from Tocris Cookson (USA) and dissolved in a vehicle consisting of 1% DMSO in pyrogen-free sterile saline (7,18). These doses were based on previous studies (7,15).

A 10-µL Hamilton syringe and a dental injection needle connected to a PE-10 tube were used to perform the microinjections into the LC of conscious rats. The injection needle was 1 mm longer than the guide cannula so that the LC was reached by the needle only at the time of injection. The microinjections were performed at a rate of 0.1 µL over a period of one minute. To prevent reflux, the needle was kept inside the guide cannula for 40 s after the end of the infusion.

### Elevated plus maze test (EPM)

EPM test was used to assess the anxiety levels of the rats. The EPM was made of wood, and consisting of two open arms (50×10 cm) crossed at right angles with two closed arms of the same size, according to the specifications described in Morato and Brandão (19). The two closed arms were enclosed by walls 50 cm high, with he exception of the central part of the maze (10×10 cm) where the open and closed arms crossed. The entire EPM was elevated 50 cm above the floor and the luminosity at the level of the EPM open arms was 30 lux. It is important to note that rats naturally avoid threatening situations represented in the model by the height and open space (19). Behavior in the EPM was recorded by a video camera linked to a monitor. This device, located outside the experimental room, allowed the recordings to be analyzed later. Rats were placed individually in the center of the maze facing a closed arm and allowed to explore the maze for a 5-min test period. EPM measures (number of entries and time spent in each arm) were recorded as described in Pellow et al. (20). The open-arm activity was evaluated as the time spent in the open arms relative to the total time spent in the plus maze (300 s), and was expressed as a percentage. At the end of the session, the rat was returned to its home cage, and before the next rat was tested, the maze was wiped clean with a 70% alcohol solution and dried with paper towels.

### Light-dark box test (LDB)

The LDB consisted of two compartments, a dark area (30 cm length×80 cm width×60 cm height) and a light area (50 cm length×80 cm width×60 cm height) connected by an opening (15 cm height×10 cm width). The box was made of plastic. The light intensity in the dark side was less than 5 lux. The light region was uncovered on the top and received room light. Rodents, which are nocturnal animals, have a natural tendency to spend more time in the dark compartment, because light places represent a natural threat. Animals were placed in the light side of the chamber facing the opening to the dark chamber and allowed to move freely between the two compartments for 5 min sessions. Behaviors were videotaped and scored using Geo Vision software. The parameters analyzed were the following: number of transitions (the number of dark compartment to light compartment transitions) and the total time spent in the light and in the dark compartments (21). An animal was considered to be in one of the compartments when its head and front paws were in that area of the box. At the end of the session, animals were returned to their home cages, and the area was wiped clean with a 70% alcohol solution.

### Experimental protocols

*Effects of intra-LC injection of ZnDPBG*. Rats that were previously cannulated were microinjected intra-LC with the HO inhibitor ZnDPBG (5, 50 or 200 nmol, 0.1 μL; n=8) or with the vehicle (Na_2_CO_3_, 50 nmol, 0.1 μL; n=6). The rats were submitted 15 min later to the EPM or the LDB test for a period of 5 min.

*Effects of intra-LC injection of heme-lysinate.* Rats that were previously cannulated were microinjected intra-LC with the HO substrate, heme-lysinate (150, 300 or 600 nmol, 0.1 μL; n=8), or with the L-lysine vehicle solution (0.1 μL; n=8). The rats were submitted 15 min later to the EPM or the LDB test for a period of 5 min.

*Effects of intra-LC injection of sGC inhibitor (ODQ)*. Rats that were previously cannulated received intracerebroventricular (*icv*) microinjections of the sGC inhibitor ODQ (1.3 nmol, 1.0 µL; n=8) or of the vehicle (DMSO 1%, 1.0 µL; n=5) followed by the intra-LC microinjection of the heme-lysinate (600 nmol, 0.1 μL; n=8) or the vehicle (L-lysine, 14.2 µmol, 0.1 μL; n=8). ODQ or its vehicle was injected in an *icv* manner to avoid multiple injections into the LC, which could eventually cause lesions in the nucleus. This method was based on a previous study (7). The rats were submitted 15 min later to the EPM or the LDB test for a period of 5 min.

### Histology

At the end of the experiments, rats were anesthetized with an intramuscular injection of ketamine (225 mg/kg, União Química Farmacêutica Nacional S.A., Brazil) plus xylasine (30 mg/kg, Carlier S.A., Spain), and transcardially perfused with saline (NaCl, 0.9%), followed by 10% formalin. The brains were then removed and fixed in 10% formalin for 4 days. The tissue was submitted to routine histological processing, and sections were observed under a microscope (Carl Zeiss model KS300, Germany) to determine the locations of the stimulated sites using the Paxinos and Watson Atlas (17).

### Statistical analysis

Results were first submitted to Levene's test for homogeneity of variance. One-way analysis of variance (ANOVA) was then used for each parameter analyzed, followed by the *post hoc* Newman-Keuls test. Data were considered statistically significant when P<0.05. Results are reported as means±SE.

## Results

**Figure 1:**
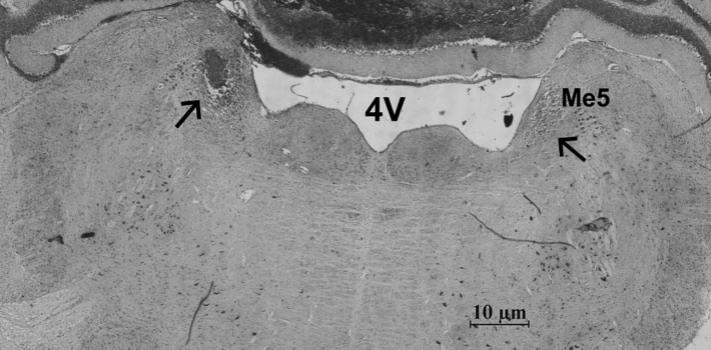
Representative photomicrograph of the right locus coeruleus (LC) of the rat. The figure shows a coronal section at the pons level illustrating the location of the intact LC (right arrow) and a typical intra-LC microinjection (left arrow). 4V: fourth ventricle; Me5: mesencephalic trigeminal nucleus.

Figure 1 shows a representative photomicrograph of the unilateral intra-LC microinjection. The microinjected area corresponds to the compact cluster of neurons adjacent to the fourth ventricle in the pontine brainstem, according to the atlas of Paxinos and Watson (17). Peri-LC microinjections caused no apparent change in EPM and LDB measures, in comparison to rats treated with vehicle in all experimental groups (data not shown).

**Figure 2:**
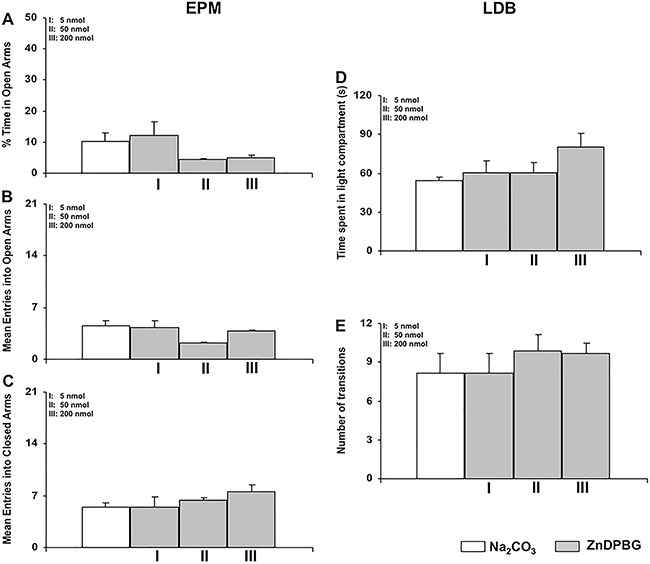
Intra-locus coeruleus administration of the nonspecific inhibitor of the enzyme heme oxygenase (HO), ZnDPBG (5, 50 and 200 nmol), or its vehicle (Na_2_CO_3_) did not alter the percentage of time spent in the open arms (*A*), the mean number of entries into open (*B*) and closed arms (*C*) in the elevated plus maze (EPM) test, and on time spent in the light compartment (*D*) and number of transitions (*E*) in the light-dark box (LDB) test. One-way ANOVA was used for statistical analysis. Data are reported as means±SE (n=6-8 animals per group).

At all doses, the intra-LC administration of ZnDPBG did not alter the percentage of time in the open arms (F=2.21, P=0.109), the mean number of entries into open arms (F=1.91, P=0.150), or the number of entries into closed arms (F=1.06, P=0.382) for rats in the EPM test (Figure 2A-C). Regarding the LDB test, intra-LC administration of ZnDPBG did not alter the time spent in the light compartment (F=1.858, P=0.161, Figure 2D), or the number of transitions in the LDB (F=0.456, P=0.715, Figure 2E).

**Figure 3:**
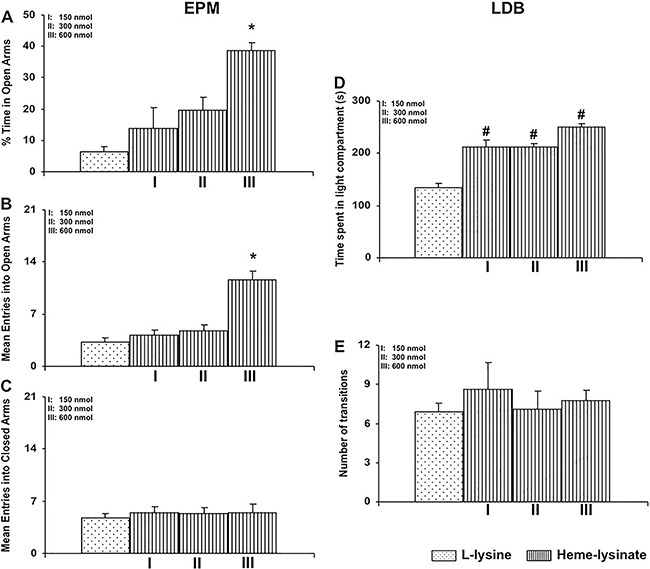
Effects of intra-locus coeruleus administration of the substrate, heme-lysinate (150, 300 and 600 nmol), or its vehicle (L-lysine) on the percentage of time spent in the open arms (*A*), the mean number of entries into open (*B*) and closed arms (*C*) in the elevated plus maze (EPM) test, and on time spent in the light compartment (*D*) and number of transitions (*E*) in the light-dark box (LDB) test. Data are reported as means±SE. *P<0.05, compared with its respective control (L-lysine) and with the 150 and 300 nmol heme-lysinate groups; ^#^P<0.05, compared with its respective control (L-lysine) (n=8 animals per group) (Newman-Keuls test).

The results of this study revealed an increase in the percentage of time spent in and entries into the open arms of the EPM by rats that received intra-LC microinjections of heme-lysinate (600 nmol, Figure 3A and B). Considering the percentage of time spent in the open arms, a significant increase between treatments was found (F=10.73, P<0.001) when comparing the 600 nmol Heme-lys group with other groups (P<0.05, Figure 3A). Regarding the mean entries into open arms, a significant increase was observed (F=20.69, P<0.001) when the 600 nmol Heme-lys group was compared with Lys and the 150 nmol and 300 nmol Heme-Lys groups (Figure 3B). Finally, the mean entries into closed arms of the EPM did not vary between treatments (Figure 3C). Regarding the LDB test, a significant increase was found (F=25.43, P<0.001) in the time spent in the light compartment for the Heme-Lys groups (150, 300 and 600 nmol) when compared with the Lys group (Figure 3D). However, Heme-Lys microinjected intra-LC, at all doses, did not alter the number of transitions of rats in the LDB (Figure 3E).

**Figure 4:**
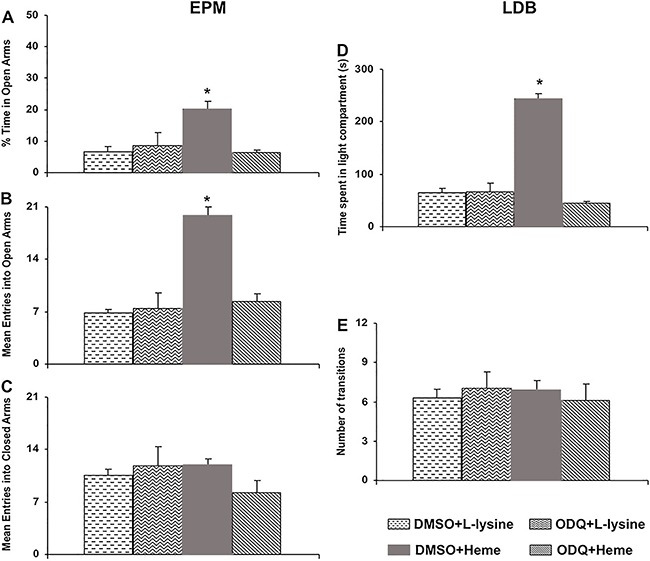
Effects of *icv* administration of 1H-(1,2,4) oxadiazolo [4,3-a] quinoxaline-1-one (ODQ) (1.3 nmol, a soluble guanylate cyclase inhibitor) or its vehicle (1% DMSO) and intra-locus coeruleus administration of the heme-lysinate (600 nmol) or its vehicle (L-lysine) on the percentage of time spent in the open arms (*A*), the mean number of entries into open (*B*) and closed arms (*C*) in the elevated plus maze (EPM) test, and on time spent in the light compartment (*D*) and number of transitions (*E*) in the light-dark box (LDB) test. Data are reported as means±SE. *P<0.05 compared to other groups (n=5-8 animals per group) (Newman-Keuls test).

The results reported in Figure 4 show that the *icv* microinjection of ODQ, an sGC inhibitor, blocked the increase in the percentage of time spent in, and entries into, the open arms of the EPM induced by intra-LC microinjections of heme-lysinate (600 nmol). Considering the percentage of time spent in the open arms, significant differences were found in the behaviors (F=61.65, P=0.002) when comparing the ODQ+Heme group with other groups (P<0.05, Figure 4A). Nevertheless, regarding the mean entries into the open arms, significant increases were observed (F=30.18, P<0.001) when comparing the ODQ+Heme group with the DMSO+L-lysine, ODQ+L-lysine and ODQ+Heme groups (Figure 4B). Moreover, the mean number of entries into the closed arms of the EPM did not vary between treatments (Figure 4C). Additionally, pre-treatment with ODQ (*icv*) blocked the increase in time spent in the light compartment (F=115.42, P<0.001) induced by the intra-LC microinjection of DMSO+Heme (600 nmol, Figure 4D) when compared with the DMSO+L-lysine, ODQ+L-lysine and ODQ+Heme groups (Figure 4D). Finally, none of the treatments altered the number of transitions in the LDB (Figure 4E).

## Discussion

The results of the present study indicated that the activity of the HO-CO pathway in the locus coeruleus can modulate anxiety, as assessed by the EPM test and the LDB test in rats. In particular, it was observed that the facilitation of enzymatic action by the administration of its substrate, heme-lysinate (at the dose of 600 nmol), promoted an increase in the number of open arm entries and the percentage of time spent in open arms in the EPM test. In a similar way, in the LDB test, it was observed that the facilitation of enzymatic action by the administration of its substrate, heme-lysinate (at the dose of 600 nmol) promoted an increase in time spent in the light compartment of the box. Taken together, these results suggest an anxiolytic effect of the HO-CO pathway in the LC. Additionally, the *icv* microinjection of the ODQ (an sGC inhibitor) followed by the intra-LC administration of heme-lysinate (600 nmol) blocked the anxiolytic-like effect on the EPM test and LDB test. It is important to note that CO has a physiological function similar to NO (9), and high levels of sGC are present in LC (8). However, NOS-like immunoreactivity is present in nuclei adjacent to LC (22). So, it is possible that increased activity of cGMP is mediated by the action of CO rather than by the action of NO in the LC (14).

There is strong evidence supporting the role of catecholaminergic LC neurons in emotional behaviors (3,23). Results from functional studies show that robust activation of the LC is observed in cats submitted to stressful situations (24), which also caused an increased expression of c-fos in this region of mice (25). In particular, a previous study (4) suggested that negative emotions, such as fear and/or anxiety, increased noradrenergic activity in the hypothalamus, amygdala and LC. Furthermore, anatomical studies demonstrated that LC projects divergent efferent pathways to the forebrain, including hypothalamus (26) and amygdala (27), structures that are essential for emotional modulation (28).

Additionally, Khoshbouei et al. (29) demonstrated that amplifying the noradrenergic response to stress by means of yohimbine treatment prior to submission to acute stress released galanin in central nucleus of the amygdala, which produced an anxiolytic response in EPM test. Considering the results obtained in this study, it is possible that the intra-LC administration of heme-lysinate increased the activity of the HO-CO pathway, which in turn could have promoted an increase of the firing rate of noradrenergic neurons of the LC (14). This could have caused an alteration of noradrenergic release in forebrain structures, such as hypothalamus and amygdala, resulting in an anxiolytic effect. Within this perspective, clinical findings suggest a relationship between the central noradrenergic system in fear/anxiety states and depression in humans. This suggestion is based on the fact that treatment with the α2-adrenergic agonist, clonidine, is effective in treating patients with anxiety disorders, whereas the administration of the α2-adrenergic antagonist exacerbates emotional symptoms (30). A previous study demonstrated that rats with chemical lesions on catecholaminergic neurons produced by a 6-hydroxydopamine injection in the LC showed normal motor activity, exploration, and habituation (23), suggesting that the LC is not essential for controlling motor activity. Corroborating previous findings, the results obtained in this study showed that heme-lysinate intra-LC administrated did not modify the mean number of entries into closed arms in EPM test, or the number of transitions in LDB test, which are parameters for locomotor behavior (2,31). So, it is possible to suggest that the anxiolytic-like effect, evidenced by an increase in the number of open arms entries and the percentage of time in open arms in the EPM test, and an increase in time spent in the light compartment in the LDB test is due to emotional modulation rather than motor activity alteration.

The noradrenergic system has been identified as one of the important regulatory systems for the hypothalamic-pituitary-adrenal axis, acting mainly on the release of corticotropin releasing factor in neurons in the paraventricular nucleus (PVN) of the hypothalamus (32). In addition, Ziegler et al. (33) showed that lesions in catecholaminergic neurons in the LC by the administration of 6-hydroxydopamine attenuated the release of ACTH in animals submitted to physical restraint stress. Considering the anatomical findings, it is possible that the activation of noradrenergic neurons of the LC promotes the inhibition of neurons in the prefrontal cortex and the disinhibition of GABAergic efferent neurons in this region, which leads to the activation of the PVN and, consequently, the activation of the hypothalamic-pituitary-adrenal circuit (34). Furthermore, the involvement of the LC in modulating states of fear and anxiety could also occur by efferent projections to the basolateral nucleus of the amygdala, because the activity of neurons in the LC promotes their own inhibition through a negative feedback (35). However, the noradrenergic modulation in the basolateral amygdala seems to have different dose-dependent effects on anxiety behavior (36). In this way, Valizadegan et al. (36) showed that low doses of salbutamol (a β-adrenergic agonist) decreased the percentage of open arms time and open arms entries in the EPM test, indicating an anxiogenic effect, while the highest dose decreased the anxiety parameters.

Initial studies conducted by Redmond et al. (37) showed that electrical stimulation of the LC in monkeys resulted in behavior observed in situations of intense fear. However, in rats, studies have shown opposite results (38,39). In particular, depletion of norepinephrine in rats resulted in increased fear and anxiety in novel places (38). On the other hand, chemical injury in the noradrenergic neurons of the LC promoted reduction of anxiogenic behaviors assessed with the EPM test, suggesting an anxiolytic effect after removing the influence of neurons in the LC (39). According to Weiss et al. (40), studies with animal models have suggested that the activity of the noradrenergic system in the LC is related to reduced anxiety and fear, contrary to a theory first described by Redmond et al. (37) in which the increased activity in neurons of the LC results in fear and anxiety. Therefore, it is clear that discrepancies exist and that more studies are still needed for a better understanding of the activity of neurons in the LC.

In summary, the results obtained in this study showed that the facilitation of enzymatic action by the administration of its substrate, heme-lysinate (at the dose of 600 nmol), promoted an increase in the number of open arm entries and the percentage of time spent in open arms in the EPM test, and an increase in time spent in the light compartment in the LDB test. This effect was blocked by the *icv* administration of ODQ, a sGC inhibitor. These data suggest that CO in the LC produced by the HO pathway and acting via cGMP played an anxiolytic role. Perhaps this anxiolytic-like effect occurred because the altered activity of the neurons of the LC, causing a potentiated noradrenergic tonus of this region, might be related to anxiety and fear modulation. Further studies are needed to clarify this involvement.
